# Effects on Rumen Microbial Population and Serum Biochemical Responses to Guanidinoacetic Acid, *Ampelopsis grossedentata* Flavonoids, and 5,6-Dimethylbenzimidazole Plus Cobalt in Lanping Black-Boned Sheep

**DOI:** 10.3390/ani15233414

**Published:** 2025-11-26

**Authors:** Zhendong Gao, Ying Lu, Huaijing Liu, Daitao Huang, Jiachen Lei, Junhong Zhu, Yuqing Chong, Weidong Deng, Jiao Wu

**Affiliations:** 1 Yunnan Provincial Key Laboratory of Animal Nutrition and Feed, Faculty of Animal Science and Technology, Yunnan Agricultural University, Kunming 650201, China; ander_gao@163.com (Z.G.); yinglu_1998@163.com (Y.L.); 18361309553@163.com (H.L.); hrynn1202@163.com (D.H.); 18792378001@163.com (J.L.); 2024022@ynau.edu.cn (J.Z.); 2022004@ynau.edu.cn (Y.C.); 2 Yunnan Rural Science & Technology Service Center, Kunming 650201, China

**Keywords:** guanidinoacetic acid, *Ampelopsis grossedentata* flavonoids, 5,6-dimethylbenzimidazole plus cobalt, rumen microbial, serum biochemical, Lanping black-boned sheep

## Abstract

To mitigate antimicrobial resistance and drug residues, the use of growth-promoting antibiotics in livestock has been prohibited, prompting interest in safe natural alternatives. Lanping black-boned sheep, an indigenous high-altitude breed in Yunnan, exhibit unique physiological traits but relatively low feed efficiency. This study evaluated the effects of three natural additives—guanidinoacetic acid (GAA, 1 g/sheep/day), *Ampelopsis grossedentata* flavonoids (AGF, 1 g/sheep/day), and 5,6-dimethylbenzimidazole plus cobalt (5,6-DMB + Co; 100 mg 5,6-DMB + 0.5 mg Co/sheep/day)—on serum biochemistry and rumen microbiota in twenty-four Lanping black-boned sheep fed under grazing conditions for 90 days following a 10-day adaptation period. GAA and 5,6-DMB + Co increased serum globulin (*p* < 0.05), whereas AGF reduced urea concentrations (*p* < 0.05). Only GAA decreased ruminal pH (*p* < 0.05). All additives enhanced microbial richness (*p* < 0.05), with 5,6-DMB + Co inducing the most pronounced community restructuring. Taxonomically, AGF and 5,6-DMB + Co decreased *Firmicutes* and enriched *Verrucomicrobiota*, while AGF uniquely increased *Cyanobacteria*. GAA selectively reduced *Quinella*, AGF decreased *Christensenellaceae_R-7_group* and *NK4A214_group*, and 5,6-DMB + Co markedly enriched *Rikenellaceae_RC9_gut_group* (*p* < 0.05). Microbial shifts were closely associated with biochemical indices, including positive associations of *Prevotella* with AST and *Rikenellaceae_RC9_gut_group* with total protein (*p* < 0.05). Overall, GAA mainly affected protein-related indices, AGF lowered serum urea, and 5,6-DMB + Co increased within-sample diversity with accompanying community reweighting, providing a theoretical basis for developing sustainable feeding strategies to enhance both productivity and health in Lanping black-boned sheep.

## Simple Summary

The Lanping black-boned sheep, a native breed from Yunnan province, China, is well adapted to high-altitude but shows relatively low feed efficiency. To improve productivity and health without relying on antibiotics, this study evaluated three natural feed additives—guanidinoacetic acid (GAA), *Ampelopsis grossedentata* flavonoids (AGF), and 5,6-dimethylbenzimidazole plus cobalt (5,6-DMB + Co). GAA increased protein-related indices, AGF was associated with lower serum urea concentration, and 5,6-DMB + Co improved microbial richness and altered community structure while promoting beneficial rumen bacterial taxa. Each additive uniquely reshaped the rumen microbiota, linking specific bacterial groups to host biochemical changes. These findings highlight that GAA, AGF, and 5,6-DMB + Co can serve as sustainable, antibiotic-free nutritional strategies to enhance both metabolic health and production performance in Lanping black-boned sheep raised under grazing conditions.

## 1. Introduction

Nutritional regulation plays a central role in improving productivity and metabolic health in ruminants, as dietary supplements can reshape rumen microbial ecosystems and influence host nutrient utilization and immune function [1]. In recent years, natural and safe feed additives have attracted increasing attention as sustainable alternatives to antibiotics, owing to their ability to modulate rumen fermentation, enhance feed efficiency, and support animal resilience [2]. With growing awareness of food safety and health, consumer demand for high-quality, residue-free meat products is increasing [3]. Although mutton is valued for its nutritional benefits, the widespread use of antibiotics in animal husbandry raises concerns over residues, antimicrobial resistance, and environmental risks [4]. To mitigate the emergence of antibiotic-resistant bacteria and drug residues, the use of growth-promoting antibiotics in livestock feed has been prohibited, prompting the search for safe and eco-friendly alternatives [5].

Lanping Black-Boned sheep, a native breed from Yunnan Province, China, are distinguished by their melanin-rich bones and tissues, strong plateau adaptability, and unique metabolic features. Genomic analyses have revealed abundant genetic diversity and a mixed ancestry with local Tibetan and lowland breeds, reflecting both adaptability and conservation potential [6,7]. Ethnobiological and physiological studies describe this breed’s traditional ecological management and its resilience to cold and hypoxic conditions at ~2500 m altitude [6]. However, despite its adaptive advantages, Lanping black-boned sheep show relatively low feed efficiency and modest meat yield. Systematic studies on its rumen microbiota, metabolic responses, or biochemical traits remain limited, underscoring the need for nutritional strategies—such as safe natural feed additives—to improve its productivity and health performance. Integrating natural feed additives offers a practical route to improve meat quality and animal health while reducing antibiotic dependence, thereby supporting more sustainable production [8,9]. Guanidineacetic acid (GAA), *Ampelopsis grossedentata* flavonoids (AGF), and the combination of 5,6-dimethylbenzimidazole with cobalt (5,6-DMB + Co) are promising natural feed additives investigated in various animal production systems. GAA, a direct precursor of creatine, supports creatine and phosphocreatine synthesis and promotes muscle energy metabolism and growth [10]. Consistent with this role, dietary GAA improved growth performance, feed efficiency, intestinal morphology, and cecal microbiota in Cherry Valley ducks [11]. Supplementing beef cattle with GAA for 140 days increased feed efficiency, enhanced serum and hepatic antioxidant indices, and reprogrammed liver protein-lipid metabolism [12]. AGF, predominantly composed of dihydromyricetin (ampelopsin), exhibits high total flavonoid content and intrinsic antioxidant capacity, and has been systematically reviewed as a promising clean-label functional food ingredient [13]. Dietary supplementation with AGF extract increased antioxidant capacity, improved immune status, reshaped the gut microbiota, and enhanced gut health in pigs [14]. Recent studies in goats and sheep have demonstrated that dietary flavonoid or polyphenol supplementation can modulate rumen fermentation, enhance microbial diversity, and improve nitrogen utilization efficiency [15,16]. Finally, the combined supplementation of 5,6-DMB and cobalt plays a critical role in the synthesis of vitamin B_12_. Insufficient cobalt intake restricts B_12_-dependent metabolism, leading to succinate accumulation and impaired fermentation [17]. Supplementation with the vitamin B_12_ precursor 5,6-DMB enhanced ruminal synthesis of true vitamin B_12_ in dairy cows, yet failed to improve its systemic availability, milk yield, or ruminal fermentation, underscoring the limited efficiency of cobalt utilization [18]. Taken together, although GAA has been extensively investigated in monogastric animals, evidence for AGF and 5,6-DMB + Co in ruminants remains scarce; moreover, studies that simultaneously supplement and compare all three additives are limited.

Gut microbiota underpins livestock performance by converting otherwise indigestible substrates into short-chain fatty acids (SCFAs), synthesizing B-vitamins, shaping bile-acid pools, and training mucosal immunity, thereby influencing nutrient harvest, feed efficiency, and disease resilience. A stable, diverse gut microbiota supports energy metabolism [19], barrier integrity and digestion through SCFAs production [20]; promotes maturation of innate and adaptive immunity while restraining inflammation [21]. In monogastrics, multiple studies show that fecal taxa such as *Lactobacillus*, *Bacteroides*, *Ruminococcus* (and related SCFA producers) associate with improved feed efficiency and carcass traits in pigs and chickens [22,23,24]. In ruminants, rumen community structure is repeatedly linked to inter-animal differences in feed efficiency, with distinct bacterial–archaeal configurations and hydrogen (H_2_) flows modulating volatile fatty acid profiles and methane losses [25,26].

The objectives of this study were, therefore, to evaluate the effects of dietary supplementation with GAA, AGF, and 5,6-DMB with cobalt on blood biochemical indices, as well as rumen microbial communities of Lanping black-boned sheep under natural grazing conditions. This research provides novel insights into the use of natural additives as viable alternatives to antibiotics, enhancing both animal health and production sustainability.

## 2. Materials and Methods

### 2.1. Ethics Statement

The study was approved by the Institutional Animal Care and Use Committee of Yunnan Agricultural University (Approval Number: 2022718, Approval Data: 8 July 2022).

### 2.2. Animals, Housing Conditions, and Experimental Design

A total of 24 healthy Lanping black-boned sheep (average age: 2 years; mean body weight: 52.91 ± 8.71 kg in Table 1) were randomly assigned to four groups (*n* = 6 per group; 3 males and 3 females), with no significant differences in initial body weight among groups (*p* > 0.05). The experiment followed a completely randomized design. Each treatment group contained three males and three females randomly assigned to ensure balanced sex distribution and comparable baseline body weight. All animals were maintained under natural grazing conditions on the same pasture and had free access to clean water. In addition to grazing, each sheep received the same batch of supplementary silage ad libitum throughout the experiment. Grazing duration, pasture access, and silage supply were identical across all treatment groups to ensure consistency in basal diet exposure.

The experiment was conducted in Lanping County, Yunnan Province, China (26°36′29″ N, 99°29′33″ E; ~2500 m above sea level). The region features a typical low-latitude mountainous monsoon climate, with a mean annual temperature of 13.7 °C and annual precipitation of 980–1007.4 mm, indicating abundant and evenly distributed rainfall. The frost-free period lasts approximately 190 d. Grassland vegetation coverage ranges from 85% to 100%, providing rich forage resources and sufficient water supply, which are favorable for sheep growth. Under these environmental conditions, the local Lanping black-boned sheep exhibit excellent adaptability and growth performance.

The experimental period consisted of a 10-day adaptation phase followed by a 90-day feeding phase. The treatments were as follows: control group (Control), GAA-supplemented group (1 g guanidineacetic acid per sheep daily, GAA group), AGF-supplemented group (1 g *Ampelopsis grossedentata* flavonoids per sheep daily, AGF group), and a 5,6-DMB + Co-supplemented group (100 mg 5,6-dimethylbenzimidazole and 0.5 mg cobalt per sheep daily, 5,6-DMB + Co group). Supplements were administered orally once daily after evening grazing using a calibrated syringe. Each additive was dissolved in clean water and gently dripped into the mouth of each sheep to ensure complete ingestion. Sheep in the control group were administered an equal volume of clean water using the same oral syringe procedure to ensure consistent handling across all groups.

### 2.3. Blood Sample Collection and Biochemical Analysis

Blood samples were collected on day 90 from the right jugular vein at 08:00 a.m. before morning grazing and feeding. To ensure fasting conditions, sheep were penned overnight without feed but with free access to water for approximately 12 h prior to sampling. Samples were centrifuged at 1500× *g* for 15 min, and the serum was separated and stored at −80 °C until analysis. Serum biochemical parameters including total protein (TP), alanine aminotransferase (ALT), aspartate aminotransferase (AST), triglyceride (TG), cholesterol (CHOL), high-density lipoprotein cholesterol (HDL-CH), low-density lipoprotein cholesterol (LDL-CH), globulin (GLOB), albumin (ALB), glucose (GLU) and urea were measured using a fully automatic biochemical analyzer (Hitachi 3100, Hitachi High-Technologies, Tokyo, Japan).

### 2.4. Rumen Fluid Collection and Microbial Analysis

Approximately 100 mL of rumen fluid was collected from each sheep on day 90 using an oral stomach tube, and the initial 20 mL was discarded to minimize salivary contamination. Samples were immediately filtered through four layers of sterile cheesecloth, and the pH was measured on-site with a digital pH meter (PHS-3C, Shanghai Instrument Co., Shanghai, China). Aliquots were snap-frozen in liquid nitrogen and stored at −80 °C until further analysis.

Microbial DNA was extracted from rumen fluid samples and sequenced on the PacBio platform (Pacific Biosciences, Menlo Park, CA, USA). Raw subreads were processed using lima (v1.7.0) and cutadapt (v1.9.1) for quality filtering, trimming, and denoising, followed by chimera removal and generation of amplicon sequence variants (ASVs) [27]. Reads were clustered into operational taxonomic units (OTUs) at 97% similarity using USEARCH [28]. Taxonomic classification was performed against the SILVA database [29]. Alpha diversity indices (ACE, Shannon, Chao1, Simpson) and β-diversity indices were calculated within QIIME2 [30]. Differentially abundant taxa were identified using LEfSe, with a linear discriminant analysis (LDA) score threshold ≥4.0 and significance set at *p* < 0.05 [31].

### 2.5. Statistical Analysis

Data were analyzed using SPSS statistical software (version 26.0, IBM Corp., Armonk, NY, USA). One-way analysis of variance (ANOVA) followed by Duncan’s multiple range test was used to identify significant differences among treatment groups. Because the male-to-female ratio was identical among groups (3:3), sex was not included as a fixed effect in the ANOVA model. Significance was defined as *p* < 0.05, and highly significant differences were defined at *p* < 0.01.

## 3. Results

### 3.1. Effects of Dietary Additives on Serum Biochemical Parameters in Lanping Black-Boned Sheep

The serum biochemical parameters of Lanping black-boned sheep in different groups are presented in Table 2. Compared with the control group, supplementation of GAA significantly increased GLOB (*p* < 0.05) and TP (*p* < 0.05) levels. Treatment with 5,6-DMB + Co significantly increased GLOB level (*p* < 0.05). AGF additive significantly reduced urea level (*p* < 0.05). Across the three additives, the GAA group showed higher urea concentrations than the AGF group (*p* < 0.05). Liver enzyme activities (AST, ALT, AST/ALT) and ALB, ALB/GLOB, TG, total cholesterol, glucose, and HDL-cholesterol did not differ among groups (*p* > 0.05), whereas AGF increased LDL-cholesterol versus Control (*p* < 0.05). In summary, the three additives induce distinct alterations in serum biochemical profiles, with GAA and 5,6-DMB + Co elevating protein-related indices and AGF affecting nitrogen and lipid parameters.

### 3.2. Effects of Dietary Additives on Rumen Fluid pH in Lanping Black-Boned Sheep

Lanping black-boned sheep in the GAA group exhibited significantly lower rumen fluid pH (6.56 ± 0.16) compared to the control group (6.80 ± 0.20, *p* < 0.05). However, the rumen fluid pH in the AGF group (6.62 ± 0.09) and the 5,6-DMB + Co group (6.66 ± 0.20) showed no significant differences compared to the control group (*p* > 0.05).

### 3.3. Dietary Additives Enhance Rumen Microbial Richness and Diversity in Lanping Black-Boned Sheep

**Figure 1:**
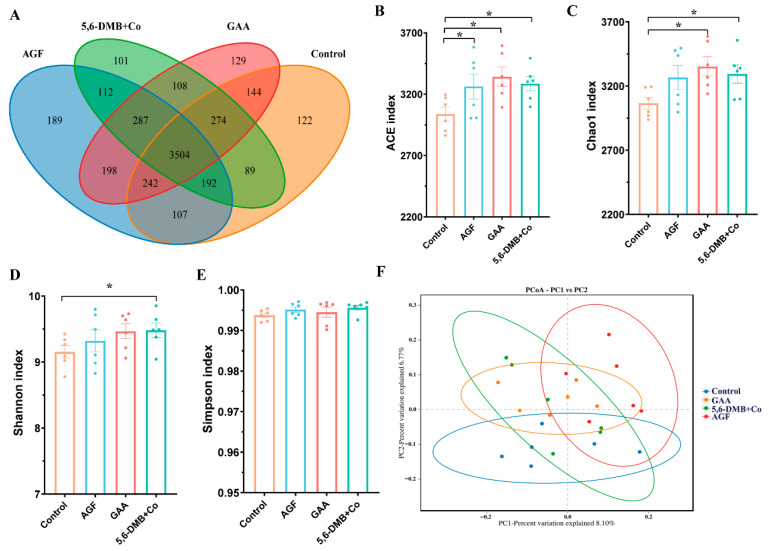
Effects of three additives on α- and β-diversity in the rumen of Lanping black-boned sheep. (**A**) Venn diagram showing the distribution of operational taxonomic units (OTUs) among the Control, GAA, AGF, and 5,6-DMB + Co groups in the rumen of Lanping black-boned sheep. (**B**–**E**) Alpha-diversity indices, including ACE (**B**), Chao1 (**C**), Shannon (**D**), and Simpson (**E**), were applied to assess community richness and diversity under different treatments. (**F**) Principal coordinates analysis (PCoA) illustrating differences in β-diversity among groups. Data are presented as mean ± SEM. *, *p* < 0.05, between each additive group and the Control.

A total of 5798 OTUs were identified in the rumen of Lanping black-boned sheep. Among them, 3504 species were shared across the Control, GAA, AGF, and 5,6-DMB + Co groups, while unique species in each group were 122, 129, 189, and 101, respectively (Figure 1A). Compared with the control group, supplementation with AGF, GAA, and 5,6-DMB + Co significantly increased the ACE index (Figure 1B, *p* < 0.05). For the Chao1 index, GAA and 5,6-DMB + Co treatments showed significant increases (Figure 1C, *p* < 0.05), whereas AGF exhibited an increasing trend without reaching statistical significance (Figure 1B, *p* > 0.05). In addition, the Shannon index was significantly elevated in the 5,6-DMB + Co group compared with the control (Figure 1D, *p* < 0.05), while the other two additives showed upward trends that were not significant (Figure 1D, *p* > 0.05). No significant effects of any of the three additives were observed on the Simpson index (Figure 1E, *p* > 0.05). PCoA of β-diversity showed distinct clustering of the 5,6-DMB + Co group from the control, indicating a pronounced shift in rumen microbiota structure, whereas GAA and AGF overlap with both the control and each other, indicating moderate effects on community composition (Figure 1F). Collectively, these results indicate that dietary supplementation with AGF, GAA, and 5,6-DMB + Co enhanced microbial richness, with 5,6-DMB + Co exerting the most pronounced impact by not only increasing diversity but also reshaping overall rumen microbial community structure.

### 3.4. Differential Remodeling of Rumen Bacterial Composition by Dietary Additives in Lanping Black-Boned Sheep

**Figure 2:**
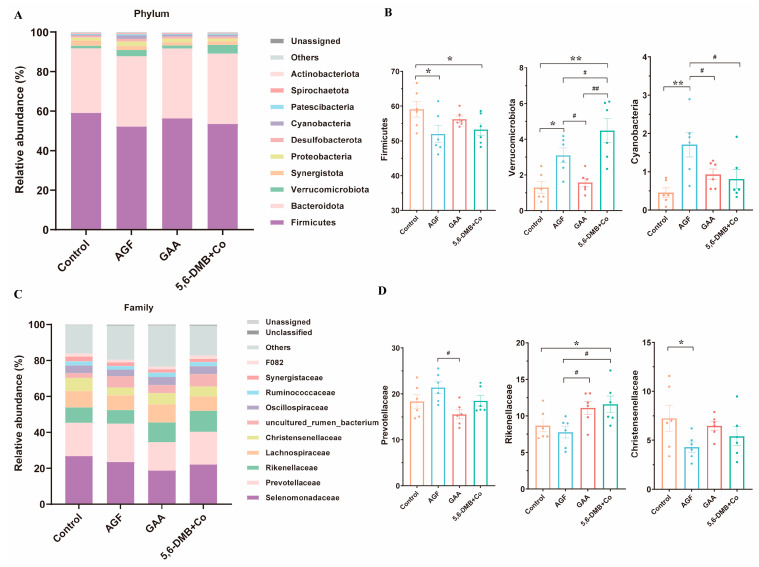
Effects of three additives on rumen microbial composition at the phylum and family levels in Lanping black-boned sheep. (**A**) Relative abundance (%) of microbial taxa at the phylum level in the Control, GAA, AGF, and 5,6-DMB + Co groups of Lanping black-boned sheep. (**B**) Phyla with significant differences, including *Firmicutes*, *Verrucomicrobiota*, and *Cyanobacteria*. (**C**) Relative abundance (%) of microbial taxa at the family level across the four groups. (**D**) Families with significant differences, including *Prevotellaceae*, *Rikenellaceae*, and *Christensenellaceae*. Data are presented as mean ± SEM. *, *p* < 0.05; **, *p* < 0.01, between each additive group and the Control; #, *p* < 0.05; ##, *p* < 0.01, among the three additive groups.

At the phylum level, the predominant bacterial communities were *Firmicutes* and *Bacteroidota* of Lanping black-boned sheep, accounting for 55.16% and 35.03% of total sequences across all groups, respectively (Figure 2A). A detailed summary of all differential taxa identified, including their taxonomic classification and relative abundances, is provided in Supplementary Table S1. Compared with the control group, supplementation with AGF and 5,6-DMB + Co significantly decreased the relative abundance of the dominant phylum *Firmicutes* (*p* < 0.05), while markedly increasing *Verrucomicrobiota* (*p* < 0.05). In addition, AGF supplementation led to a highly significant increase in *Cyanobacteria* abundance (*p* < 0.01). Comparisons between three additives revealed stronger enrichment of *Verrucomicrobiota* under 5,6-DMB + Co than AGF and GAA, whereas *Cyanobacteria* showed opposite responses to AGF and 5,6-DMB + Co (Figure 2B). At the family level (Figure 2C), the predominant bacterial taxa in the rumen of Lanping black-boned sheep were *Selenomonadaceae* (22.58%), *Prevotellaceae* (18.44%), *Rikenellaceae* (9.77%), and *Lachnospiraceae* (8.88%). Compared with the control group, supplementation with 5,6-DMB + Co markedly increased the abundance of *Rikenellaceae* (*p* < 0.05), with a stronger effect than that observed for AGF or GAA. In addition, AGF supplementation significantly reduced the abundance of *Christensenellaceae* (*p* < 0.05), whereas GAA significantly decreased *Prevotellaceae* among three additives (*p* < 0.05).

**Figure 3:**
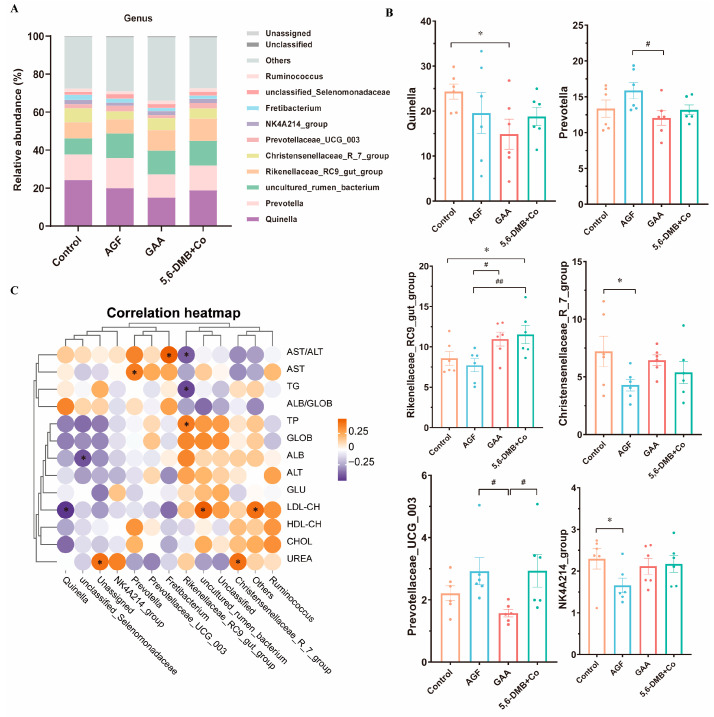
Effects of three additives on rumen microbial composition at the genus level and correlations with serum biochemical parameters in Lanping black-boned sheep. (**A**) Relative abundance (%) of microbial taxa at the genus level in the Control, GAA, AGF, and 5,6-DMB + Co groups of Lanping black-boned sheep. (**B**) Genera with significant differences, including *Quinella*, *Prevotella*, *Rikenellaceae_RC9_gut_group*, *Christensenellaceae_R-7_group*, *Prevotellaceae_UCG_003*, and *NK4A214_group*. (**C**) Heatmap of correlations between rumen microbial genera and serum biochemical indices. *, *p* < 0.05, indicates significant correlations. Data are presented as mean ± SEM. *, *p* < 0.05, between each additive group and the Control; #, *p* < 0.05; ##, *p* < 0.01, among the three additive groups.

At the genus level, the predominant bacterial taxa in the rumen of Lanping black-boned sheep were *Quinella*, *Prevotella*, *uncultured_rumen_bacterium*, and *Rikenellaceae_RC9_gut_group* (Figure 3A). Compared with the control group, GAA supplementation significantly reduced the abundance of *Quinella*. Among the three additives, GAA also significantly decreased *Prevotella* and *Prevotellaceae_UCG_003*. In contrast, AGF supplementation significantly lowered the abundances of *Christensenellaceae_R-7_group* (*p* < 0.05) and *NK4A214_group* (*p* < 0.05) relative to the control. Notably, 5,6-DMB + Co supplementation significantly increased the abundance of *Rikenellaceae_RC9_gut_group* compared with the control (*p* < 0.05), and this effect was stronger than that observed for AGF and GAA (Figure 3B). In a word, all three additives remodel the rumen microbiota in Lanping black-boned sheep. GAA preferentially depletes and *Quinella*; AGF reduces *Firmicutes* while expanding *Verrucomicrobiota* and *Cyanobacteria* and suppressing *Christensenellaceae/NK4A214_group*; and 5,6-DMB + Co likewise lowers *Firmicutes* but more strongly increases *Verrucomicrobiota* and markedly enriches *Rikenellaceae_RC9_gut_group*, exerting the opposite effect on *Cyanobacteria* to AGF. Taken together, these findings demonstrate that each additive uniquely reshaped the rumen bacterial community, highlighting additive-specific ecological niches and compositional responses in Lanping black-boned sheep.

### 3.5. Associations Between Rumen Bacterial Genera and Serum Biochemical Parameters in Lanping Black-Boned Sheep

Rumen microbiota composition correlates with serum biochemistry in Lanping black-boned sheep. Significant associations (*p* < 0.05) included positive links of *Prevotella* with AST and *Fretibacterium* with AST/ALT, a positive link of *Christensenellaceae_R-7_group* with urea, and for *Rikenellaceae_RC9_gut_group* a positive link with TP but negative links with AST/ALT and TG; *Quinella* correlated negatively with LDL-CH, whereas *uncultured_rumen_bacterium* and others correlated positively with LDL-CH, and unassigned correlated positively with urea (Figure 3C). These results indicate that additive-induced, genus-level reconfiguration aligns with host serum biochemistry.

### 3.6. Differential Bacterial Biomarkers Identified by LEfSe Analysis in Lanping Black-Boned Sheep

**Figure 4:**
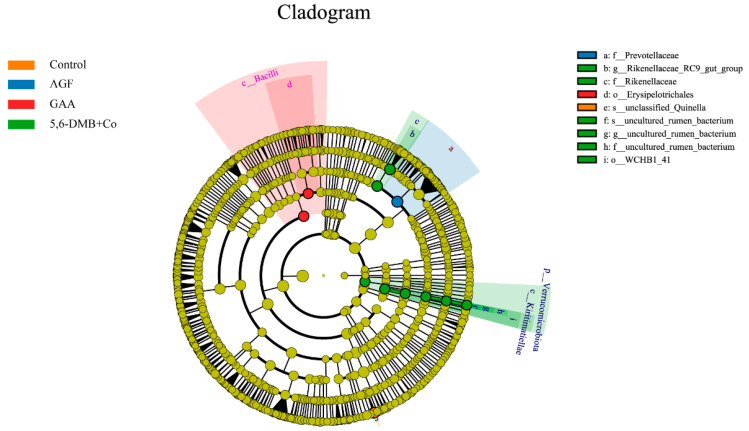
LEfSe analysis of differentially enriched rumen bacterial taxa among the three additive groups in Lanping black-boned sheep. Cladogram showed the phylogenetic distribution of bacterial lineages significantly enriched in the Control, GAA, AGF, and 5,6-DMB + Co groups. Colored nodes represent taxa with significant differences, and circle size is proportional to relative abundance.

LEfSe analysis (Figure 4) revealed that *c _Bacilli* and *o_Erysipelotrichales* were significantly enriched in the GAA group. The 5,6-DMB + Co group exhibited the most pronounced microbial shifts, with significant enrichment of *g_Rikenellaceae_RC9_gut_group*, *f_Rikenellaceae*, *p_Verrucomicrobiota*, *c_Kiritimatiellae*, *o_WCHB1_41*, as well as several uncultured rumen bacteria across different taxonomic levels. In contrast, the AGF group was characterized by enrichment of *f_Prevotellaceae*, whereas the control group showed a predominance of *s_unclassified_Quinella*. Collectively, these results indicate that different additives induced distinct ruminal bacterial profiles in Lanping black-boned sheep.

## 4. Discussion

The effects of dietary supplementation with GAA, AGF, and the combined supplementation of 5,6-DMB + Co on blood physiological and biochemical indices and microbial community structure in Lanping black-boned sheep were evaluated. According to the results, each additive exerts distinct impacts on host metabolism and rumen microbial ecology.

GAA and 5,6-DMB + Co supplementation significantly increased serum globulin concentrations, with GAA also elevating total protein levels, indicating an association with enhanced immune-related indicators. This aligns with previous reports indicating that GAA, as a creatine precursor, spares arginine for protein and nitric oxide synthesis, thus supporting protein accretion and immune competence in ruminants [32]. These findings are consistent with recent studies that report coordinated shifts in microbiota and serum metabolome under GAA supplementation, although the effects on other serum variables can be context-dependent and vary across studies [33]. In contrast, AGF supplementation markedly reduced serum urea concentrations, indicating improved nitrogen utilization efficiency. This result corroborates earlier studies suggesting that flavonoids can suppress ruminal proteolysis and deamination, thereby enhancing nitrogen retention [34]. The reduction in urea concentration is consistent with other reports that demonstrate the role of flavonoids in modulating rumen microbial communities and improving nitrogen efficiency, especially in caprine models [16]. The concomitant increase in LDL-cholesterol under AGF supplementation suggests a possible trade-off in lipid metabolism, which aligns with the broader literature indicating that plant polyphenols can influence hepatic lipid metabolism and elevate plasma LDL levels through antioxidant modulation and gene expression regulation [35].

Similarly, the reduction of rumen pH by GAA, indicative of intensified fermentation, was within a physiological range and is in line with previous studies showing that GAA supplementation can enhance rumen fermentative activity and VFA production in other ruminants like Hu sheep [36]. While VFA concentrations were not measured in the current study, the observed pH reduction could reflect similar fermentative shifts as those reported in the literature, which warrants further investigation through direct VFA quantification in future studies.

The microbial richness increase observed with all three additives—GAA, AGF, and 5,6-DMB + Co—was consistent with findings from previous studies showing that natural additives can enhance microbial diversity and community structure in the rumen [37]. Interestingly, the 5,6-DMB + Co supplementation significantly increased Shannon diversity and altered microbial β-diversity, demonstrating a strong reshaping effect on the microbial ecosystem. This is in agreement with previous studies highlighting the ecological role of cobamides and their impact on rumen microbial communities, as many microbes are auxotrophic for vitamin B_12_ and rely on community exchange of corrinoids [38]. These findings suggest that enhancing cobamide precursors can promote cross-feeding, broaden ecological niches, and restructure microbial communities, supporting the hypothesis that cobamides can modulate ruminal fermentation [37,38].

At the phylum level, both AGF and 5,6-DMB + Co reduced *Firmicutes* and increased *Verrucomicrobiota*, indicating a shift toward microbes specialized in mucin and polysaccharide degradation. This pattern agrees with reports that plant-derived metabolites can enrich *Verrucomicrobiota* (especially *Akkermansia*) and reduce *Firmicutes* after flavonoid supplementation [39,40,41]. The suppression of *Christensenellaceae* by AGF, a family associated with host leanness and lipid metabolism regulation, mirrors similar findings from studies that show plant polyphenols influence lipid metabolism and microbiota composition in livestock [42]. This pattern may be mechanistically linked to the reduced abundance of *Christensenellaceae*, reflecting altered bile-acid signaling or hepatic lipid turnover, thereby increasing circulating LDL-CH. Such lipid–nitrogen trade-offs are consistent with previous studies showing that plant polyphenols and citrus flavonoids can modulate hepatic lipid metabolism, improve antioxidant capacity, and transiently elevate plasma LDL levels during metabolic adaptation [43,44,45]. Therefore, AGF may influence both nitrogen and lipid metabolism through coordinated microbial–host interactions, rather than direct lipid synthesis pathways.

At the genus level, the suppression of *Prevotella* and *Quinella* by GAA in this study aligns with findings from other research indicating that these genera are key players in peptide turnover and carbohydrate fermentation in the rumen [46,47]. Similarly, AGF-driven suppression of *Christensenellaceae_R-7_group*, linked to lipid metabolism, echoes findings from studies on citrus peel extract, which demonstrated changes in *Christensenellaceae* abundance and improved lipid metabolism in dairy cows [48]. The *NK4A214_group*, a fiber-associated *Ruminococcaceae* lineage, often fluctuates with diet composition and short-term adaptation rather than showing a uniform response to polyphenols [49]. The changes in the microbial community under 5,6-DMB + Co, including the increase in *Rikenellaceae_RC9_gut_group*, were also similar to those reported in other cobalt-based studies, highlighting that cobalt’s effects may vary depending on the additive matrix and dose [50].

Our correlation analysis further supports the functional consequences of microbial shifts observed in this study, linking them to serum biochemical indices. For example, *Prevotella*’s positive correlation with AST reflects earlier studies in beef cattle that highlighted its role as a microbial indicator of rumen fermentation intensity and hepatic metabolic responses [51]. Similarly, *Quinella*’s negative correlation with LDL-cholesterol is consistent with its role in regulating lipid metabolism within the rumen [52]. The positive correlation between *Rikenellaceae_RC9_gut_group* and total protein, and its negative association with AST/ALT and triglycerides, is in line with findings from previous research that suggested a role for this group in protein metabolism and hepatic function [53]. These correlations highlight that microbial community shifts are not just compositional but have functional implications for host metabolism, reinforcing the importance of microbial–host interactions in livestock nutrition.

It should be noted that the absence of direct ruminal fermentation parameters (e.g., volatile fatty acids, ammonia-N) represents a limitation of this study. Therefore, some interpretations of microbial–metabolic interactions remain speculative and should be validated in future studies combining fermentation profiles with metabolomic or transcriptomic analyses. To improve statistical robustness, future studies should include larger cohorts, as our sample size per group (*n* = 6), although comparable to previous ruminant microbiota studies, may limit the characterization of inter-individual variation. To improve the characterization of potential sex-dependent responses, future studies should include larger sex-stratified cohorts, as the present sample size per sex (*n* = 3) was insufficient for modeling sex as a fixed factor. The absence of pre-treatment serum and rumen microbiota measurements represents a limitation, as baseline variation cannot be fully excluded. Future studies should incorporate day-0 sampling to more clearly distinguish treatment effects from initial inter-individual differences. The absence of chemical analysis of the basal forage and silage is a limitation of this study. Future work should incorporate representative sampling to improve dietary characterization and reproducibility. Given that sampling was performed only once at the end of the 90-day feeding period, short-term temporal dynamics could not be assessed. However, the terminal measurements reflect stabilized steady-state responses following prolonged exposure, which is consistent with the study’s objective to evaluate endpoint physiological and microbial adaptations under grazing conditions.

**Figure 5:**
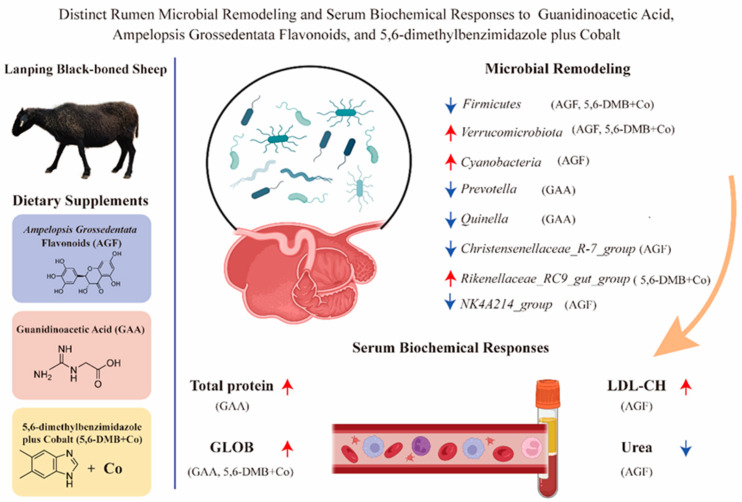
Distinct rumen microbial remodeling and serum biochemical responses to guanidinoacetic acid, Ampelopsis grossedentata flavonoids, and 5,6-dimethylbenzimidazole plus cobalt in Lanping black-boned sheep. Red upward arrows indicate an increase, whereas blue downward arrows indicate a decrease.

Taken together, our results indicate that each additive exhibits distinct advantages and limitations. GAA was associated with enhanced immune indicators by increasing serum globulin and total protein but concomitantly reduced glycolytic taxa such as *Prevotella* and *Quinella*. AGF suggested improved nitrogen utilization efficiency through reduced serum urea levels while reshaping the rumen microbiota by decreasing *Firmicutes* and *Christensenellaceae* and expanding *Verrucomicrobiota* and *Cyanobacteria*, though accompanied by elevated LDL-cholesterol. 5,6-DMB + Co had the most pronounced impact, significantly enhancing microbial diversity and enriching *Rikenellaceae_RC9_gut_group*, which aligned with improved protein metabolism and serum biochemical profiles (Figure 5). These findings provide mechanistic insights into the host–microbe interactions shaped by nutritional additives and highlight the potential of tailoring additive use to specific production goals in Lanping black-boned sheep.

## 5. Conclusions

In conclusion, dietary supplementation with AGF, GAA, and 5,6-DMB + Co was associated with additive-specific shifts in serum chemistry and rumen bacterial composition of Lanping black-boned sheep. GAA mainly affected protein-related indices, AGF lowered serum urea, and 5,6-DMB + Co increased within-sample diversity with accompanying community reweighting. These results provide a theoretical basis for developing sustainable feeding strategies to enhance both productivity and health in Lanping black-boned sheep.

## Figures and Tables

**Table 1:** Baseline characteristics of Lanping black-boned sheep used in the experiment.

Group	*n*	Sex (M/F)	Body Weight (kg, Mean ± SD)	Notes
Control	6	3/3	50.55 ± 12.78	Healthy, randomly assigned
GAA	6	3/3	52.03 ± 8.35	GAA (1 g/d)
AGF	6	3/3	52.03 ± 6.61	AGF (1 g/d)
5,6-DMB + Co	6	3/3	51.52 ± 6.05	5,6-DMB (100 mg/d) + Co (0.5 mg/d)

**Table 2:** Effects of three additives on serum biochemistry parameters in Lanping black-boned sheep.

Item	Control	AGF	GAA	5,6-DMB + Co
GLOB (g/L)	45.13 ± 5.49 ^b^	50.56 ± 6.44 ^ab^	53.90 ± 4.18 ^a^	52.24 ± 5.55 ^a^
ALB (g/L)	25.83 ± 3.87	25.24 ± 2.14	27.56 ± 2.24	26.29 ± 1.96
ALB/GLOB	0.57 ± 0.05	0.52 ± 0.04	0.50 ± 0.00	0.52 ± 0.00
TP (g/L)	70.96 ± 8.64 ^b^	75.79 ± 8.36 ^ab^	81.46 ± 6.07 ^a^	78.52 ± 6.39 ^ab^
UREA (mmol/L)	8.05 ± 1.51 ^a^	6.54 ± 0.61 ^b^	7.87 ± 0.79 ^a^	7.25 ± 0.80 ^ab^
AST (U/L)	150.95 ± 39.60	147.08 ± 41.54	117.60 ± 12.42	159.90 ± 44.25
ALT (U/L)	22.80 ± 3.73	22.25 ± 4.46	20.92 ± 5.75	23.45 ± 2.51
AST/ALT	6.74 ± 1.89	6.75 ± 2.16	6.11 ± 2.30	6.97 ± 2.40
TG (mmol/L)	0.28 ± 0.05	0.29 ± 0.05	0.27 ± 0.03	0.25 ± 0.04
CHOL (mmol/L)	1.71 ± 0.19	1.86 ± 0.19	1.66 ± 0.22	1.77 ± 0.24
GLU (mmol/L)	3.28 ± 0.53	3.15 ± 0.36	3.53 ± 0.88	3.39 ± 0.31
HDL-CH (mmol/L)	1.08 ± 0.15	1.09 ± 0.16	0.95 ± 0.18	1.06 ± 0.14
LDL-CH (mmol/L)	0.47 ± 0.06 ^b^	0.58 ± 0.06 ^a^	0.55 ± 0.06 ^ab^	0.54 ± 0.09 ^ab^

Note. Control = without additive; GAA = guanidinoacetic acid; AGF = *Ampelopsis grossedentata* flavonoids; 5,6-DMB + Co = 5,6-dimethylbenzimidazole plus cobalt. Data are presented as mean ± SD (*n* = 6 per group). That letter indicates significant difference (*p* < 0.05), while the same letter indicates no significant difference (*p* > 0.05).

## Data Availability

All the data are accessible.

## Supplementary Materials

The following supporting information can be downloaded at: https://www.mdpi.com/article/10.3390/ani15233414/s1, Table S1. Differential bacterial taxa identified among treatment groups (Control, GAA, AGF, and 5,6-DMB + Co), including phylum, family, and genus levels, along with relative abundances.
